# Identification of regulatory SNPs associated with genetic modifications in lung adenocarcinoma

**DOI:** 10.1186/s13104-015-1053-8

**Published:** 2015-03-24

**Authors:** Tzu-Pin Lu, Chuhsing K Hsiao, Liang-Chuan Lai, Mong-Hsun Tsai, Chung-Ping Hsu, Jang-Ming Lee, Eric Y Chuang

**Affiliations:** Department of Public Health, Institute of Epidemiology and Preventive Medicine, National Taiwan University, Taipei, Taiwan; Bioinformatics and Biostatistics Core, Center of Genomic Medicine, National Taiwan University, Taipei, Taiwan; Graduate Institute of Physiology, National Taiwan University, Taipei, Taiwan; Institute of Biotechnology, National Taiwan University, Taipei, Taiwan; Division of Thoracic Surgery, Taichung Veterans General Hospital, Taichung, Taiwan; Department of Surgery, National Taiwan University Hospital, Taipei, Taiwan; Graduate Institute of Biomedical Electronics and Bioinformatics and Department of Electrical Engineering, National Taiwan University, Taipei, Taiwan

**Keywords:** Lung adenocarcinoma, Integrated analysis, Copy number variation, Methylation, Microarray, SNP

## Abstract

**Background:**

Although much research effort has been devoted to elucidating lung cancer, the molecular mechanism of tumorigenesis still remains unclear. A major challenge to improve the understanding of lung cancer is the difficulty of identifying reproducible differentially expressed genes across independent studies, due to their low consistency. To enhance the reproducibility of the findings, an integrated analysis was performed to identify regulatory SNPs. Thirty-two pairs of tumor and adjacent normal lung tissue specimens were analyzed using Affymetrix U133plus2.0, Affymetrix SNP 6.0, and Illumina Infinium Methylation microarrays. Copy number variations (CNVs) and methylation alterations were analyzed and paired *t*-tests were used to identify differentially expressed genes.

**Results:**

A total of 505 differentially expressed genes were identified, and their dysregulated patterns moderately correlated with CNVs and methylation alterations based on the hierarchical clustering analysis. Subsequently, three statistical approaches were performed to explore regulatory SNPs, which revealed that the genotypes of 551 and 66 SNPs were associated with CNV and changes in methylation, respectively. Among them, downstream transcriptional dysregulation was observed in 9 SNPs for CNVs and 4 SNPs for methylation alterations.

**Conclusions:**

In summary, these identified SNPs concurrently showed the same direction of gene expression changes with genetic modifications, suggesting their pivotal roles in the genome for non-smoking women with lung adenocarcinoma.

**Electronic supplementary material:**

The online version of this article (doi:10.1186/s13104-015-1053-8) contains supplementary material, which is available to authorized users.

## Background

Lung cancer has become an important cause of cancer-related death in the United States, Europe, and worldwide [1-3]. Although countless research efforts have been devoted to understanding the etiology of lung cancer, the tumorigenesis process still remains unclear. An important reason to this is the low reproducibility of the identified differentially expressed genes across independent lung cancer cohorts. Consequently, even if many prognostic biomarkers have been identified for predicting survival outcomes for lung cancer patients [4-6]; their application is usually limited due to the low reproducibility [7,8]. Various confounding factors, including heterogeneous sample characteristics, different experimental procedures, and multiple statistical algorithms, may lead to this inconsistency. Since changes at DNA-level are more stable than that at RNA-level, one possible strategy to improve the reproducibility is to perform integrated analyses of gene expression and genome modifications, such as copy number variations (CNVs) and methylation alterations.

It is well-known that DNA copy number is a causative factor in driving downstream gene expression changes, especially in cancer tissues. A previous study has demonstrated that approximately 12% of gene expression changes can be explained by the concordant CNVs in breast cancer [9]. In addition, a genome-wide approach has revealed many recurrent CNVs in lung adenocarcinoma [10], and an integrated analysis has reported that several functionally relevant gene sets were successfully utilized as prognostic biomarkers for at least three independent cohorts [11]. Therefore, with the advancement in high-resolution karyotyping technologies, such as microarrays and next-generation sequencing, researchers are able to investigate genomic landscapes of CNVs *en masse* at a lower cost. A comprehensive analysis of concurrent CNVs and gene expression patterns may help to provide better understanding and identify important dysregulated genes for lung cancer.

DNA methylation is a common modification mechanism in the regulation of normal physiological function, and gene expression levels may be affected through altered methylation profiles [12]. Recently, a growing body of evidence has indicated that changes in DNA methylation play an important role in tumor initiation and progression [12-14], perhaps by reactivating oncogenes that are normally silenced. Conversely, locus-specific hypermethylation usually occurs in tumor suppressors and thus leads to their loss of function. For example, methylation of *CDKN2A*, a major player in cell cycle regulation, was suggested as a potential biomarker of lung cancer due to its existence in pre-neoplastic lesions in smokers, but not non-smokers [15]. In addition to regional methylation alterations, global hypomethylated patterns in tumor tissue were associated with the progression of lung cancer [15]. Thus, an integrated analysis of gene expression and methylation alterations may help to improve the understanding of gene regulation mechanisms in lung cancer.

High-throughput technologies facilitate the screening of hotspots for CNVs or methylation alterations in cancer studies. However, their use in practice poses a major problem in that a relatively low frequency of such genomic changes may be reported. For instance, the most commonly observed amplification in lung adenocarcinoma, 14q13.3, was only observed in 12% of samples [10]. To address this issue, several studies have indicated that some of those genetic modifications were accounted for by *cis*-acting regulatory elements [16-18]. Such regulation mechanisms with allelic asymmetries were widespread in non-imprinted single nucleotide polymorphisms (SNPs) and were associated with not only CNVs but also methylation alterations in many cancer studies [17-19]. Compared with SNPs identified from genome-wide association screenings, these regulatory SNPs showing functional relevance have clearer roles in cancer development. In addition, the differential preferences of tumor cells for specific SNP alleles have been reported in several cancers [17-19], suggesting germline alleles are important regulators in driving downstream gene expression. Therefore, taking this hereditary effect into account may provide a better understanding of lung cancer etiology.

In this study, we performed an integrated genome-wide association study with CNVs and methylation alterations in non-smoking lung adenocarcinoma women. These two genetic modification mechanisms were correlated with downstream gene expression changes, and several SNP loci were closely associated with the dysregulated expression patterns. Differential expression analyses of the transcription level of these SNP loci identified nine alleles associated with CNVs and three alleles associated with methylation alterations. Among them, those SNPs showing concordant changes (i.e., simultaneous and in the same direction) in both gene expression and genetic modification may serve as potential candidates for further experimental validation in lung adenocarcinoma.

## Methods

### Sample collection and microarray experiments

Thirty-two pairs of lung tumor and adjacent normal tissue specimens from non-smoking female adenocarcinoma patients were collected from National Taiwan University Hospital or Taichung Veterans General Hospital. These 32 patients are a subset of the lung cancer patients examined in our previous studies [6,11] and all of them belong to adenocarcinoma subtype. The study protocol was conducted in accordance with the Declaration of Helsinki and was approved by the local ethics committees. All samples were concurrently analyzed using Affymetrix SNP 6.0, Illumina Infinium Methylation, and Affymetrix U133plus2.0 microarrays. The extraction of DNA and RNA was performed following standard protocols provided by the manufacturers. The mean ± SD age of the patients was 62 ± 10 years, and 78% (25/32) were in stage I or II. These and other patient characteristics are summarized in Additional file 1: Table S1 and further details about the information of sample collection and clinical features is provided in the previous study [6]. The microarray data have been submitted to Gene Expression Omnibus with the accession numbers of GSE19804 [6], GSE33355 [20], and GSE49996.

### Microarray data analyses

For gene expression analysis, tumor and adjacent normal tissues from the same individual were investigated using an Affymetrix U133plus2.0 microarray. After quality checks, raw intensity data were imported into Partek Genomic Suite for analysis. Quantile normalization was performed to remove systematic bias. Subsequently, paired *t*-tests were utilized to identify genes expressed differentially in tumor and normal tissue.

Genome-wide SNPs were assessed using an Affymetrix SNP 6.0 array, which contains 906,600 probes in total. Among all examined SNPs, only those loci with a minor allele frequency of at least 0.01 and in Hardy-Weinberg equilibrium (*p*-value > 0.05) were retained for further analysis.

In addition to examining the genotyping results, the intensity data of each SNP probe were imported into Partek software to perform CNV analysis. Since both tumor and adjacent normal tissues from the same individual were investigated, the reference baseline for each tumor tissue is its own corresponding normal tissue. Genomic segments were defined if they met the following criteria: minimum consecutive genomic markers ≥ 100, *p*-value ≤ 0.001, and signal-to-noise ratio (SNR) ≥ 0.3. Among the identified segments, only the regions with absolute copy number changes of at least 0.3 were considered copy number variation regions (CNVRs), i.e., the copy number of an amplified region was higher than 2.3, and the copy number of a deleted region was lower than 1.7. The genes located within or overlapping with these detected CNVRs were annotated by the documentation file provided by Affymetrix.

Whole-genome methylation profiles were analyzed with the Illumina Infinium Methylation Assay. There were 27,578 methylation probes, which contained approximately 20,000 distinct CpG islands and 14,000 unique genes. Similar to the above gene expression and CNV analyses, paired comparisons were performed to identify genes with unequal methylation levels. The threshold of beta value difference between tumor and normal tissue was defined as 0.25 based on a previous study [21]. That is, hypermethylated regions were defined if there was a positive difference in beta values (≥0.25), and hypomethylated regions if there were negative differences (≤ −0.25).

### Associations among copy number, methylation, and gene expression

**Figure 1:**
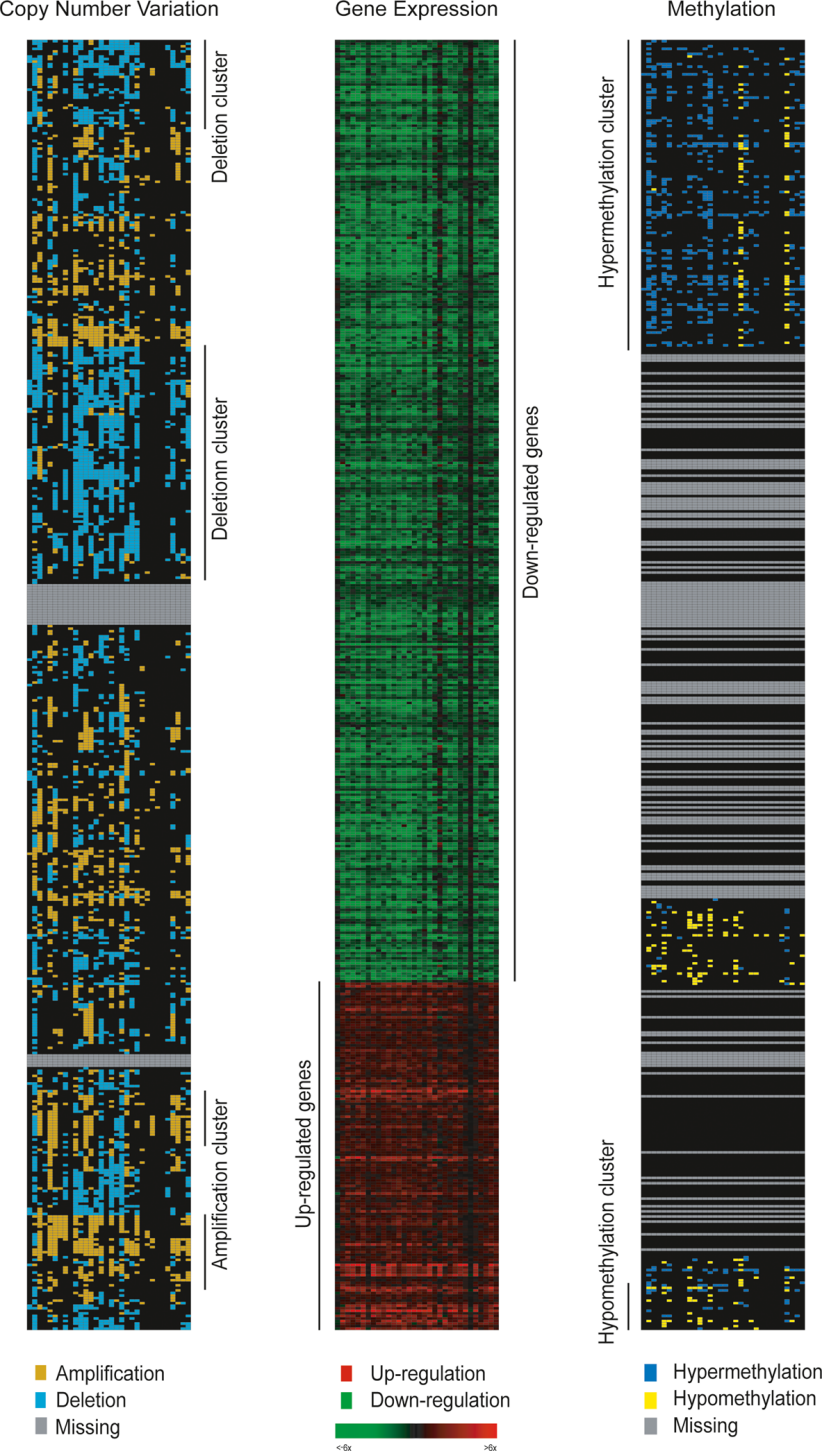
**Heatmaps of gene expression, copy number variation (CNV), and methylation level of the 505 differentially expressed genes in tumor tissue.** In the gene expression heatmap (middle column), red represents up-regulation and green represents down-regulation. The gene order was the same in all three panels. For CNV and methylation alteration (left, right columns), one-way hierarchical clustering was first performed on the β-value difference and subsequently the copy number difference. For CNV (left column), gold indicates amplification and cyan indicates deletion. For methylation level (right column), blue denotes hypermethylation and yellow denotes hypomethylation.

To explore the associations among CNVs, methylation, and gene expression levels, hierarchical clustering was performed using the Genesis program [22]. The input data were the gene expression ratios between tumor and normal tissue of the differentially expressed genes (Figure 1, middle column). Using the same order of genes, corresponding CNVs and methylation changes in those genes were illustrated as heatmaps (Figure 1, left and right panels, respectively).

### Identification of SNP loci associated with copy number variations

Our procedure for identifying regulatory SNPs associated with different CNVs in lung tumor and adjacent normal tissues is outlined in a flowchart (Additional file 1: Figure S1). For each gene in every sample, DNA copy number status was defined as amplified or deleted, if the magnitude of its copy number difference between tumor and normal tissue was larger than 0.3 (i.e., amplified: CN_dif_ ≥ 0.3; deleted: CN_dif_ ≤ −0.3). For each SNP in a normal tissue, two coding schemes, considering the qualitative effects or quantitative effects, were used to classify patients into different groups. In the qualitative effect model, a SNP variable of a sample was coded as “0” or “1” for carrying the corresponding allele or not; in the quantitative effect model, a SNP variable was coded as “2”, “1,” or “0” to evaluate if the qualitative effect was additive. To explore the associations between CNVs and SNP classifications, three statistical approaches were performed under each model. First, for every SNP in all samples, Fisher’s exact test was applied to a 2×2 or 3×2 contingency table to evaluate whether its specific groupings were correlated with CNVs (Additional file 1: Tables S2 and S3). Subsequently, linear regression models (Additional file 1) were utilized to estimate if the difference in copy number between tumor and normal tissue was predicted by the SNP variable. Lastly, a Kruskal-Wallis test was performed to examine the median differences in copy number among various SNP groups, and notably the Kruskal-Wallis test is equivalent to the Wilcoxon rank sum test while the patients are classified into two groups.

### Identification of SNP loci associated with methylation alterations

In addition to CNVs, the same procedures shown in Additional file 1: Figure S1 were performed to examine methylation alterations. For every gene with changes in methylation, patients were divided into two groups: (a) the “hypermethylated” group, in which the beta value differences between tumor and normal tissue were higher than 0.25, and (b) the “hypomethylated” group, in which the beta value differences between tumor and normal tissue were lower than −0.25. Similar to the above copy number analysis , samples were classified based on two SNP coding schemes, and the associations between these specific groups and methylation alterations were evaluated with Fisher’s exact test, linear regression models, and a Kruskal-Wallis test.

## Results

### Dysregulated patterns among methylation alterations, copy number variations, and gene expression changes

The whole genome expression profiles of 32 non-smoking women with lung adenocarcinoma were examined using Affymetrix U133 plus2.0 expression arrays. Since both cancerous and adjacent normal tissues from the same individual were investigated, a paired *t*-test was used to identify differentially expressed genes. Five hundred five genes showed significant changes with *p*-values < 10^−9^, and this criterion was more stringent than the threshold obtained by Bonferroni correction (0.05/54675 = 9.14 × 10^−7^). The expression changes of the 505 genes are illustrated by a hierarchical clustering heatmap (Figure 1, middle column). Among them, 369 genes (73%) were down-regulated and 136 genes (27%) were up-regulated in tumor tissues, a distribution comparable to our previous study [6].

To explore possible genetic regulation mechanisms of these dysregulated genes, CNV analysis was performed with Affymetrix SNP 6.0 arrays, and whole genome methylation profiles were assessed with Illumina Infinium Methylation arrays. For CNV analysis, genes were defined as amplified or deleted if the magnitude of copy number changes was greater than 0.3; for methylation analysis, genes were defined as hypermethylated or hypomethylated if the magnitude of methylation alterations was greater than 0.25. (The detailed procedures of identification of CNVs and methylation alterations were described in Materials and Methods.) To visualize these genetic modifications with their corresponding transcriptional changes, one-way hierarchical clustering was performed first on differences in methylation level and subsequently on CNVs (Figure 1, left and right columns). Although some genes showed CNV and gene expression changing in opposing directions, about 60% of genes demonstrated co-varying patterns, especially the amplification and deletion clusters (Figure 1, left column). In addition to CNVs, methylation alterations were associated with gene expression dysregulations, especially those simultaneously hypermethylated and down-regulated genes in the upper half (Figure 1, right column). These results suggest that CNVs and methylation alterations both play important roles in modulating downstream transcriptional changes, and concurrent explorations of them will help to elucidate the regulation mechanisms in lung adenocarcinoma. To obtain biomarkers for predicting these genetic modifications in lung tumorigenesis, the following analyses focused on regulatory SNPs with functional relevance to them.

### Regulatory SNPs associated with genetic modifications including copy number variations and methylation alterations

To identify potential regulators of CNVs and methylation alterations, whole genome SNPs were investigated using Affymetrix SNP 6.0 arrays. After excluding those SNPs not in Hardy-Weinberg equilibrium (*p*-values < 0.05) and with minor allele frequency lower than 0.01, 511,263 SNPs were collected for further analysis. For each SNP, two coding schemes, the “qualitative model” and the “quantitative model,” were used and patients were divided accordingly into different groups based on their genotyping results. The qualitative effect model considered whether the existence of a specific SNP allele is correlated with genetic modifications, and the quantitative effect model evaluated if there existed additive associations. To take the above two models into account simultaneously, we designed a flowchart including three statistical approaches (Addional file 1: Figure S1).

**Figure 2:**
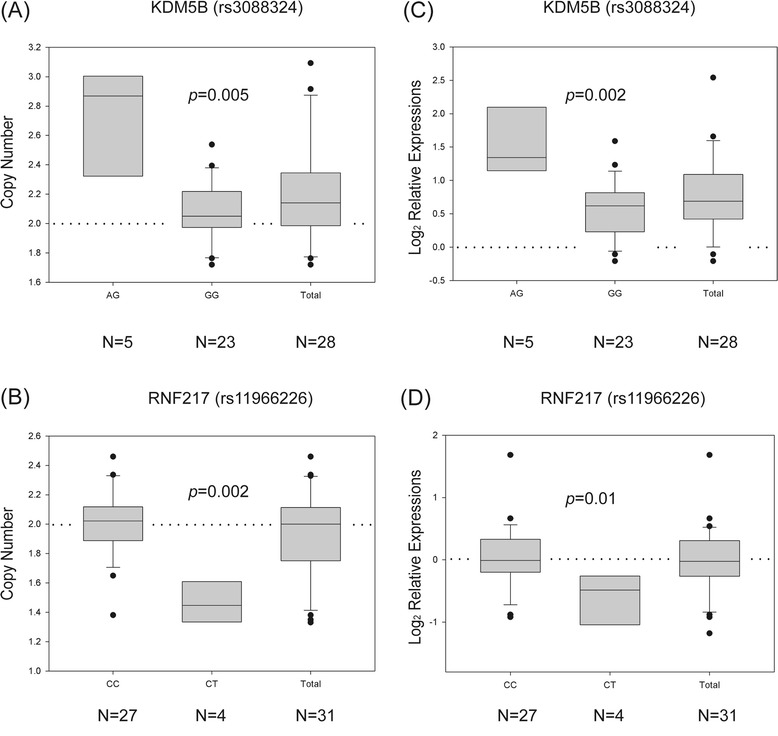
**Boxplots of two SNPs, rs3088324 and rs11966226, showing significant association with copy number variation. (A-B)** Boxplots were used to demonstrate the difference in copy number between the specific SNPs (AG versus GG for rs3088324 and CC versus CT for rs11966226). Significance level was determined by a Wilcoxon rank sum test. The Y-axis represents copy number. **(A)** *KDM5B*, rs3088324, was correlated with copy number amplifications. **(B)** *RNF217*, rs11966226, was correlated with copy number deletions. **(C-D)** To evaluate whether CNVs were able to drive downstream gene expression changes, a Wilcoxon rank sum test was performed on the expression difference between tumor and normal tissue for each SNP. The two genes, **(C)** *KDM5B* and **(D)** *RNF217*, both showed significantly differential expression (*p*-values < 0.05) and concordance in terms of the direction of change of CNVs and gene expression. The Y-axis denotes relative expression ratios on a log scale. Dotted lines indicate the unchanging baseline.

First, Fisher’s exact test was used to explore the relationships between CNVs and SNP groupings; i.e., for each SNP in the two models, a 2×2 or 3×2 contingency table was created and evaluated. As shown in Table 1, there were 1,048 and 843 SNPs showing significant associations with copy number amplifications or deletions, respectively (*p*-values < 0.01). Among these SNPs, a linear regression test was applied to examine whether the copy number differences were predicted by the coding variables in each model. The results showed that 551 SNPs successfully predicted the magnitude of CNVs with *p*-values of < 0.01, indicating that the correlations between these SNPs and CNVs were both qualitative and quantitative. Subsequently, a Kruskal-Wallis test was used to evaluate the classification performances of the groupings from those SNPs, which revealed 142 and 95 SNPs with significant copy number differences (*p*-values < 0.01). For example, rs3088324 in *KDM5B* was associated with amplifications, and rs11966226 in *RNF217* was associated with deletions (Figure 2A-B). These results indicate that investigation of SNPs and CNVs concurrently may help to identify dysregulated hotspots of genetic amplifications/deletions. To explore whether those CNVs were able to trigger downstream gene expression changes, a Kruskal-Wallis test was performed on their expression differences according to SNP groupings. A few SNPs were significantly (*p*-values < 0.05, Table 2) associated with corresponding changes in transcription level, such as *KDM5B* and *RNF217* (Figure 2C-D), which further demonstrated that explorations of CNVs based on SNPs may reveal important hereditary markers during lung tumorigenesis.

**Table 1:** **Number of significant SNPs in three statistical approaches including Fisher’s exact test, linear regression, and Kruskal-Wallis test**

**Genetic modification**	**Fisher’s exact test** ^**a**^	**Linear regression** ^**a**^	**Kruskal-Wallis test** ^**a**^
Copy number variations			
Amplification	1048	362	142
Deletion	843	189	95
Methylation alterations			
Hypermethylation	316	39	28
Hypomethylation	120	27	17

^a^ *p*-value threshold was set as 0.01 in three tests.

**Table 2:** **List of SNPs showing differential expression changes based on the SNP groupings**

**Genetic modification**	**Gene symbol**	**SNP**	**Concordance** ^**a**^
**Copy number variations**			
**Amplification**	*KDM5B*	rs3088324	+
	*SSBP2*	rs4703852	+
	*SMYD3*	rs4654180	+
	*TRPC7*	rs4976373/rs7727634	-
	*SEMA6A*	rs11951689	+
**Deletion**	*RNF217*	rs11966226cu	+
	*PCDH8*	rs9596724	-
	*NAMPT*	rs41799	-
**Methylation alterations**			
**Hypermethylation**	*MAGI2*	rs17150656	+
	*MYOM2*	rs6558659/rs2405327	-
	*FGF12*	rs9881554	-
**Hypomethylation**	---	---	---

^a^Concordant (+) or discordant (−) changes in gene expression and genetic modification.

**Figure 3:**
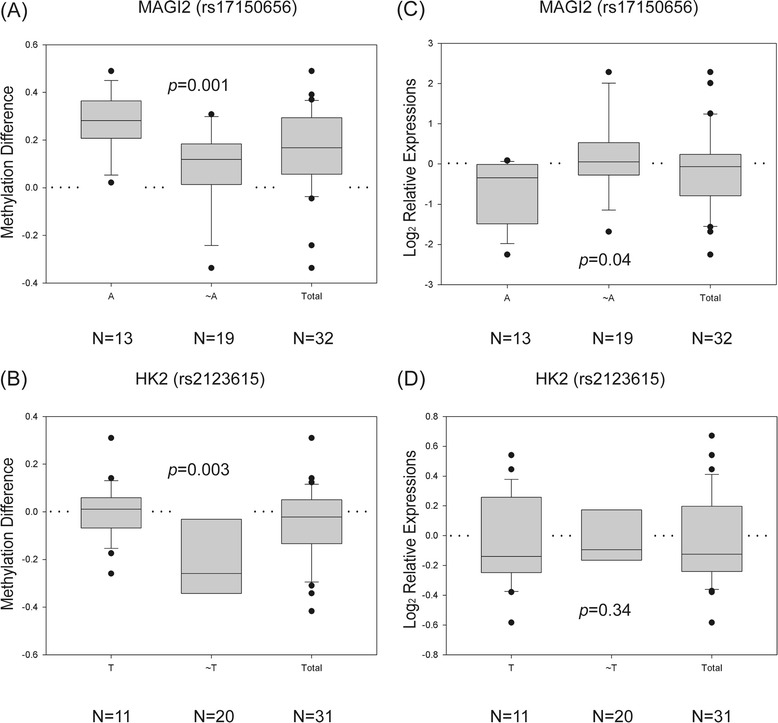
**Boxplots of two SNPs, rs17150656 and rs2123615, showing significant association with methylation alterations. (A-B)** Boxplots were used to demonstrate the difference of methylation level between specific SNPs (AA and AG versus GG for rs17150656 and TT and TG versus GG for rs2123615). Significance level was determined by a Wilcoxon rank sum test. The Y-axis represents methylation differences. **(A)** *MAGI2*, rs17150656, was correlated with hypermethylation. **(B)** *HK2*, rs2123615, was correlated with hypomethylation. **(C-D)** To evaluate whether the methylation alterations were able to drive downstream gene expression changes, a Wilcoxon rank sum test was performed on the expression difference between tumor and normal tissue for each SNP. **(C)** *MAGI2* showed significantly differential expression (*p*-values < 0.05) but **(D)** *HK2* did not. The Y-axis denotes relative expression ratios on a log scale. Dotted lines indicate the unchanging baseline.

In addition to CNVs, the relationships of SNPs and methylation alterations were examined by the same procedures described previously. There were 316 and 120 SNPs showing associations with hypermethylation and hypomethylation, respectively, according to Fisher’s exact test (Table 1). Among them, linear regression models indicated that 66 SNPs successfully predicted the magnitude of methylation alterations. A Kruskal-Wallis test further excluded 21 SNPs due to insignificant differences among their specific groupings, which resulted in 28 and 17 SNPs associated with hyper- and hypomethylations, respectively. These results indicate that certain SNPs might be able to trigger methylation alterations during lung tumorigenesis. For instance, rs17150656 in *MAGI2* was related to hypermethylation, and rs2123615 in *HK2* was related to hypomethylation (Figure 3A-B). Similar to the analysis of CNVs, gene expression changes corresponding to these methylation alterations were examined by Kruskal-Wallis tests. Four SNPs with relevance to hypermethylation showed significantly differential expression among the specific groupings, such as *MAGI2* (Figure 3C), but no such SNP was identified from the pool relevant to hypomethylation (Table 2). Compared with the results from CNVs, fewer SNPs associated with methylation alteration were regarded as significant loci in driving downstream transcription changes. However, those SNPs still deserved further investigation, since dysregulated patterns showing concordance in the direction of change of both methylation alterations and gene expression were observed.

## Discussion

In previous studies, several prognostic biomarkers for lung cancer have been identified based on gene expression microarrays. However, further clinical applications of them often resulted in extremely low reproducibility across independent studies. This may be attributed to the fact that transcriptional changes related to lung cancer tumorigenesis and progression are contributed by many complicated factors, such as CNVs and changes in methylation [10,15]. To better elucidate the dysregulated cancer genome in lung tumorigenesis, we performed an integrated analysis in non-smoking lung adenocarcinoma patients to identify SNP alleles associated with CNVs and methylation alterations. Gene expression analysis further demonstrated that several SNP alleles with genetic modifications correlated with downstream transcriptional changes. By comparing these identified SNP loci with differentially expressed genes from gene expression microarrays, we may observe lower false-positive rates of biomarker identification by limiting the search to changes that are consistent across three levels—sequence, epigenomic, and transcriptional—of the cancer genome in lung adenocarcinoma.

Gene expression analysis identified 505 differentially expressed genes in 32 lung adenocarcinoma patients, and most of them were down-regulated in tumor tissue. Approximately 70% of these genes were identical to those observed in 60 women with non-small cell lung cancer in our previous study [6]. Although a higher proportion of significantly down-regulated genes was observed (Figure 1), the number of up-regulated genes in the whole genome was comparable to that of down-regulated genes (Additional file 1: Figure S2). This result is dependent on the threshold selected for significance, which was *p* < 10^−9^ in this study.

Regarding the associations with CNVs and methylation alterations, it is obvious that more genes showed changes in copy number than in methylation level (Figure 1). However, there were many genes (40%) showing discordance between CNVs and gene expression changes. The reason for such inconsistent change remains unclear, and it might imply the existence of other regulatory mechanisms with antagonistic effects at the transcription level. Therefore, the more genomic changes considered in the gene expression analysis, the better performance exhibited by the results.

Compared with CNVs, fewer genes with methylation alterations were demonstrated (Figure 1), which may be attributed to the fact that current methylation microarray technologies mainly focus on promoter regions and CpG islands. Recent reports have shown that several methylated loci were in regulatory regions outside CpG islands or core promoters [23,24]. For example, methylation changes occurred in sequences whose nearest promoter or CpG island is 2 kb away in a colon cancer study [23]. This suggests that experimental methodologies with higher resolution, such as next-generation sequencing, are required to improve the detection performance. In our study, the patterns of methylation alteration correlated moderately with corresponding gene expression changes, consistent with methylation alterations being able to drive downstream gene expression in cancer cells [23]. We conclude that incorporating such epigenetic modifications into transcriptional analysis may help to identify genes differentially expressed in tumorigenesis with lower false discovery rates.

To classify patients into different groups based on the SNP genotyping results, germline alleles in adjacent normal tissue were utilized rather than those in tumor tissue. In general, highly similar results were obtained in both analyses, since only a few SNPs were different between tumor and normal tissues. Those SNPs may correspond to somatic mutations in lung tumorigenesis that preferentially amplify oncogenic alleles in tumor cells. To evaluate whether there were any SNPs showing significant changes, Bowker’s test was performed and indicated that no such varied allele was identified with *p*-value of < 0.05. Furthermore, another important reason for choosing normal tissue as a reference baseline was that these SNPs represent hereditary effects and may serve as possible biomarkers for lung tumorigenesis in advanced applications.

**Figure 4:**
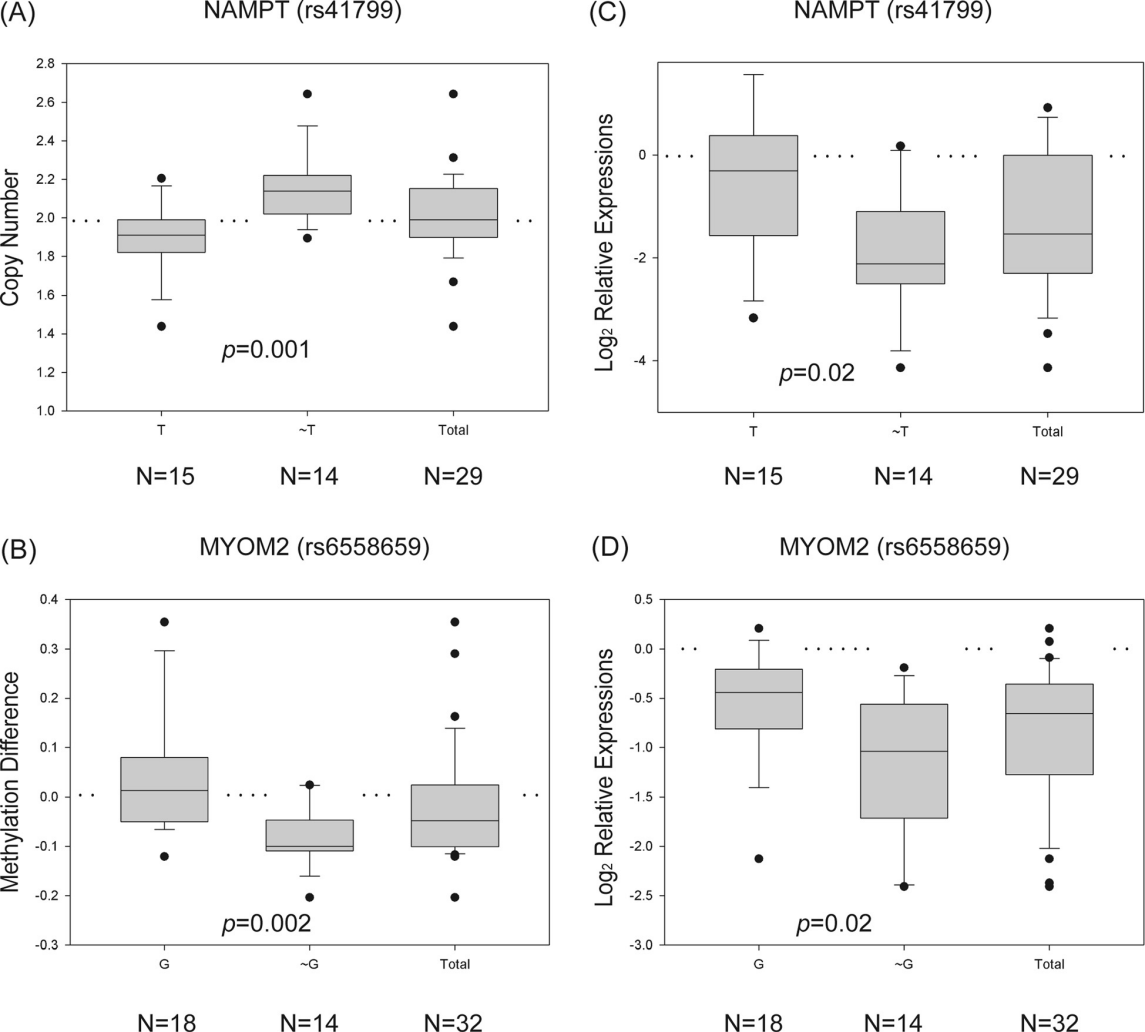
**Boxplots of two SNPs, rs41799 and rs6558659, showing discordant changes between DNA modification and gene expression. (A-B)** Boxplots were used to demonstrate the difference in copy number **(A)** or methylation level **(B)** between the specific SNPs (TT and TG versus GG for rs41799 and GG and GT versus TT for rs6558659). Significance level was determined by a Wilcoxon rank sum test. The Y-axes represent copy number **(A)** or methylation difference **(B). (A)** *NAMPT*, rs41799, was correlated with amplification. **(B)** *MYOM2*, rs6558659, was correlated with hypomethylation. **(C-D)** To evaluate whether the genetic modifications were able to drive downstream gene expression changes, a Wilcoxon ranksum test was performed on the expression difference between tumor and normal tissue for each SNP. **(C)** *NAMPT* and **(D)** *MYOM2* both showed significantly differential expression (*p*-values < 0.05), but in the opposite direction with respect to the corresponding DNA modification. The Y-axis denotes relative expression ratios on a log scale. Dotted lines indicate the unchanging baseline.

Many SNP alleles were correlated with CNVs or methylation alterations (Table 1), but only thirteen of them showed downstream gene expression changes (Table 2). Among these 13 SNPs, some showed methylation and transcription changes in the opposite direction (Figure 4). This low penetration rate from genetic variation to transcription could result from those SNPs being identified by random chance, or alternatively, from other as-yet uncharacterized mechanisms participating in gene regulation, such as transcription factor and microRNA activity or histone acetylation. Tumor tissues could use these additional mechanisms to antagonize the dysregulation effects driven by CNVs and methylation if they alter the essential genes for tumor development (i.e., amplifications or hypomethylations on tumor suppressors and deletions or hypermethylations on oncogenes). Therefore, those SNPs without concordant transcriptional changes deserve further investigation to clarify their roles in lung adenocarcinoma.

Among those SNPs with concordant genetic modifications and transcriptional changes (Table 2), some have been reported in previous studies to serve as important players in cancer cells [25-29]. For instance, knockdown of *SMYD3* induced apoptosis through G1-phase cell cycle arrest in breast cancer cell line MDA-MB-231 [26], and *MAGI2* cooperates with *PTEN* to inhibit the growth of tumor cells by suppressing the activation of Akt [28]. *KDM5B*, also known as *JARID1B*, was required to maintain tumorigeneic activity in melanoma cells [29], and the knockdown of *KDM5B* may trigger apoptosis and reduces proliferation in bladder and lung cancers [25]. Therefore, ongoing efforts are warranted to further elucidate the roles these identified SNPs play in lung tumorigenesis.

## Conclusions

An integrated analysis of gene expression, SNPs, CNV, and methylation alteration was performed in 32 non-smoking women with lung adenocarcinoma. Moderate correlations were demonstrated between genetic modifications and gene expression levels, indicating that concurrent analysis of DNA and RNA levels may improve the homogeneity of the findings. The integrated protocol proposed in this study revealed unidirectional transcriptional changes in 9 SNPs for CNVs and 4 SNPs for methylation alterations. Further functional studies are warranted to elucidate the biological roles of these SNP-gene combinations in lung adenocarcinoma.

## Additional file

Additional file 1:
**Supplementary Tables and Figures.**

## Abbreviations

CNV: Copy number variation
CNVR: Copy number variation region
SNP: Single nucleotide polymorphism
