# Supplementary material for: Identification of regulatory SNPs associated with genetic modifications in lung adenocarcinoma
Source: BMC Res Notes. 2015 Mar 24;8:92. doi: 10.1186/s13104-015-1053-8 (PMC4384239; doi:10.1186/s13104-015-1053-8)
Supplement: Additional file 1: — Supplementary Tables and Figures. [file 13104_2015_1053_MOESM1_ESM.pdf]

Supplementary materials

Supplementary Tables

Table S1. Sample characteristics of lung cancer patients examined by Affymetrix SNP6.0, Illumina Infinium methylation, and Affymetrix U133plus 2.0 microarrays.

Characteristics	Microarray
**Sample Number**	32
**Age**	62.2±10
**Histology**	
**Adenocarcinoma**	32 (100%)
**Gender**	
**Female**	32 (100%)
**Stage**	
**IA**	7 (22%)
**IB**	11 (34%)
**IIA**	2 (6%)
**IIB**	5 (16%)
**IIIA**	4 (13%)
**IIIB**	2 (16%)
**IV**	1 (3%)

Table S2. Example of Fisher's exact test in one SNP locus of qualitative effect.

SNP	Amplified	Unchanged
A	0	29
~A	2	1

Table S3. Example of Fisher's exact test in one SNP locus of quantitative effect.

SNP	Amplified	Unchanged
AA	0	14
AB	0	15
BB	2	1

Supplementary Figures

Figure S1. Flowchart for regulatory SNP identification. Two SNP coding schemes were utilized for comparison. The first one was “qualitative effect,” which explored whether the existence of allele A or allele B played an important role in driving downstream methylation changes or copy number variations. In addition, the other one was “quantitative effect,” which took the numbers of allele A or allele B into the statistical models. Detailed procedures of the statistical approaches were described in the “Materials and Methods” section.

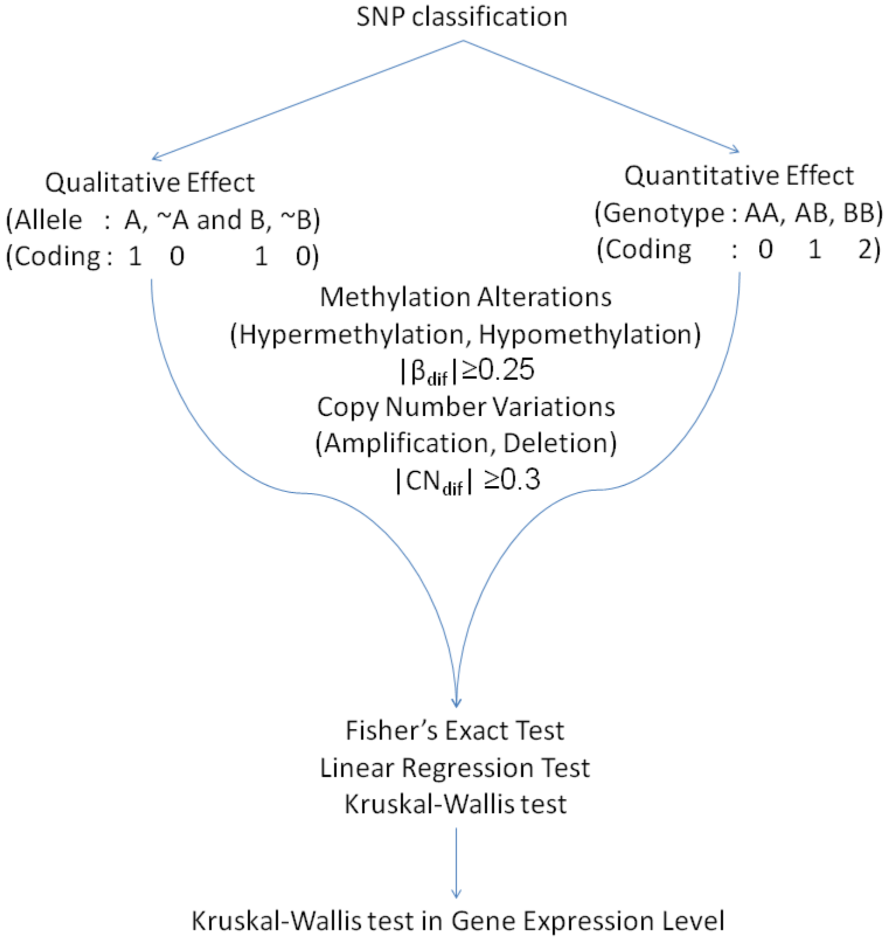

Figure S2. Volcano plot of all genes in the Affymetrix U133plus2.0 microarray. The –log (p-value) of each gene is plotted against the log2 ratio of cancer intensity to normal intensity. The yellow line indicates the significance level at $p = 10^{-9}$. Red dots indicate genes that were up-regulated in cancer tissues; green dots indicate down-regulated genes.

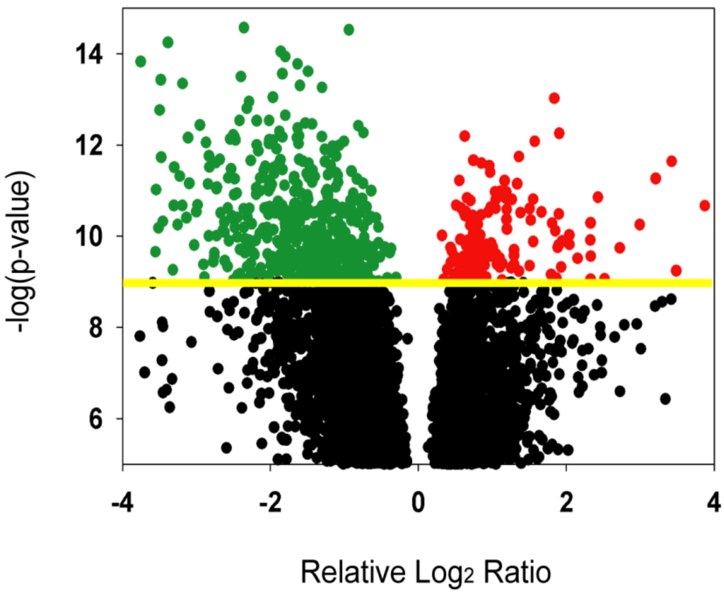

Formulas

Formula 1. Regression model of the association between a SNP locus and genomic variations when considering SNP qualitative effects.

$$y_{ij} = x_{ij} * \beta + \text{Constant}$$

$$\begin{Bmatrix} x_{ij} = 1, & \text{if allele i in sample j} = \text{AA, AB} \\ x_{ij} = 0, & \text{if allele i in sample j} = \text{BB} \end{Bmatrix} \text{ or } \begin{Bmatrix} x_{ij} = 1, & \text{if allele i in sample j} = \text{AB, BB} \\ x_{ij} = 0, & \text{if allele i in sample j} = \text{AA} \end{Bmatrix}$$

where x_{ij} denotes whether the SNP allele i exists or not and y_{ij} denotes for gene i in sample j the methylation beta value difference between tumor and normal tissues after log transformation. For CNV analysis, y_{ij} denotes the copy number difference between tumor and normal tissues for gene i in sample j.

Formula 2. Regression model of the association between genotype coding of one SNP locus and genomic variations when considering SNP quantitative effects.

$$y_{ij} = x_{ij} * \beta + \text{Constant}$$

$$\left\{\begin{array}{ll} x_{ij} = 0, & \text{if SNP i in sample j} = \text{AA} \\ x_{ij} = 1, & \text{if SNP i in sample j} = \text{AB} \\ x_{ij} = 2, & \text{if SNP i in sample j} = \text{BB} \end{array}\right\}$$

where x_{ij} denotes the genotype coding for one SNP and y_{ij} denotes for gene i in sample j the methylation beta value difference between tumor and normal tissues after log transformation. For CNV analysis, y_{ij} denotes the copy number difference between tumor and normal tissues for gene i in sample j.
